# Oral administration of soybean okara extract reduces osteoarthritis pain and progression in rats

**DOI:** 10.7150/ijms.123212

**Published:** 2026-01-01

**Authors:** Kun-Tsan Lee, Chien-Min Chen, Chih-Hsin Tang, Yen-You Lin, Tzu-Ching Chang, Min-Huan Wu, Sunny Li-Yun Chang

**Affiliations:** 1 Department of Post-Baccalaureate Medicine, National Chung-Hsing University, Taichung, Taiwan.; 2 Department of Orthopedics, Taichung Veterans General Hospital, Taichung, Taiwan.; 3 Sinying Hospital, Ministry of Health and Welfare, Tainan, Taiwan.; 4 Department of Pharmacology, School of Medicine, China Medical University, Taichung, Taiwan.; 5 Chinese Medicine Research Center, China Medical University, Taichung, Taiwan.; 6 Department of Medical Laboratory Science and Biotechnology, College of Medical and Health Science, Asia University, Taichung, Taiwan.; 7 Graduate Institute of Biomedical Sciences, China Medical University, Taichung, Taiwan.; 8 Translational Medicine Center, Shin Kong Wu Ho-Su Memorial Hospital, Taipei, Taiwan.; 9 Master in Senior Heath and Exercise Sciences, Tunghai University, Taichung, Taiwan.; 10 Department of Physiology, School of Medicine, College of Medicine, China Medical University, Taichung, Taiwan.

**Keywords:** soybean okara, antioxidant, anti-inflammatory agents, osteoarthritis

## Abstract

Osteoarthritis (OA) is a common inflammatory degenerative disease that causes joint pain and irreversible bone damage, affecting many middle-aged and elderly people. Currently, there is no effective treatment available. Okara, a byproduct of soybean processing, contains bioactive compounds with antioxidant and anti-inflammatory properties similar to those found in soybeans, making it a promising candidate for reuse as a food supplement to provide health benefits. In this study, we explored the therapeutic potential of soybean okara extract (SOE) in OA using a rat surgical anterior cruciate ligament transection (ACLT) OA model. Oral administration of SOE significantly alleviated bone pain associated with ACLT, as demonstrated by a weight-bearing behavioral assay. Histopathological analysis revealed that oral SOE ameliorated ACLT-induced bone destruction, improved cartilage and synovium integrity, and reduced the levels of proinflammatory cytokines IL-1β, TNF-α, and the chondrolytic enzyme MMP-3. This, in turn, led to a decrease in the degradation of aggrecan and collagen type II, thereby preserving cartilage. These findings suggest that oral administration of SOE could be a promising approach for the prevention and treatment of OA.

## Introduction

With advances in medicine and increased life expectancy, degenerative diseases have become some of the most common health challenges, with osteoarthritis (OA) being among the most prevalent. According to the Global Burden of Disease report, approximately 528 million individuals worldwide were living with OA, representing a 114.5% increase in prevalence between 1990 and 2019 1. The pathological features of OA include subchondral bone sclerosis, cartilage degradation, and synovial tissue inflammation, which are often irreversible at the time of diagnosis and result in joint pain and stiffness 2, 3. OA is currently incurable, with treatment options limited to slowing disease progression or alleviating pain.

Chronic inflammation of the synovial tissues is closely associated with joint pain and the development of structural damage and with synovial release, playing an essential role in driving inflammation and tissue destruction in OA 4, 5. The elevated expression of proinflammatory cytokines, such as interleukin (IL)-1β and tumor necrosis factor (TNF)-α, along with synovium-secreted mediators, such as matrix metalloproteinase (MMP)-3, is markedly correlated with the severity of knee OA and may be associated with OA progression 6-9. Relevant studies indicate that anti-inflammatory approaches are a potential therapeutic strategy for OA 4, 10.

Soybean, a widely consumed plant-based protein source, is rich in essential nutrients, comprising approximately 35-40% protein, 20% lipids, 9% dietary fiber, and 8.5% moisture 11. Soybeans are abundant in bioactive compounds, including isoflavones, saponins, and bioactive peptides and proteins (such as the Bowman-Birk protease inhibitor) 11-14. The consumption of soybeans has been associated with a reduced risk of various chronic diseases, including obesity, cardiovascular disease, neurodegenerative disorders, and osteoporosis 9, 12. Among the isoflavones, genistein and daidzein, which together constitute about 90% of soybean-derived isoflavones, have been extensively reported for their health benefits. Notably, genistein has demonstrated protective effects against bone and cartilage-related disorders, including OA, rheumatoid arthritis, osteoporosis, and intervertebral disc degeneration 15.

Soybeans, due to their low cost and high nutritional value, are processed into various products, such as soy milk, tofu, firm tofu, and tofu skin, which have been integral to Asian diets for millennia. This processing results in the production of large quantities of soybean residue, known as okara. Okara retains many of the nutrients found in soybeans, including bioactive isoflavones, albeit in different proportions 16, 17. Recent studies have reported that oral consumption of okara can attenuate cognitive decline, mirroring the preventive effects of soy isoflavones against cognitive deficits and Alzheimer's disease 16, 18-20. Given its nutritional composition, similar to that of soybeans, okara presents a valuable opportunity for repurposing as a nutritious food supplement, contributing to sustainable food production 21.

The high moisture content and rich nutritional profile of raw okara contribute to its short shelf life, typically limited to a few days under refrigeration. In this study, we aimed to extend the shelf life of okara by preparing dried okara using an oven dryer. Additionally, we investigated the protective effects of soybean okara extract (SOE) against OA in a preclinical rat model induced by anterior cruciate ligament transection (ACLT). We focused on the analysis of key inflammatory mediators, such as IL-1β, TNF-α, and the chondrolytic enzyme MMP-3, which play crucial roles in OA progression 22. Our findings demonstrate that SOE significantly reduces inflammation and offers protective effects against OA in the ACLT-induced rat model.

## Materials and Methods

### Materials

Antibodies for immunohistochemistry were obtained from various suppliers: IL-1β (MAB601) from R&D Systems, Inc. (Minneapolis, MN, USA); TNF-α (A11534) and collagen II (A1560) from ABclonal, Inc. (Woburn, MA, USA); MMP-3 (SC-21732) from Santa Cruz Biotechnology (Dallas, TX, USA); and aggrecan (ab3778) from Abcam (Cambridge, UK). Other chemicals and reagents were sourced from Sigma-Aldrich (St. Louis, MO, USA).

### Preparation of Soybean Okara Extract

Okara was obtained from Maido Co., Ltd, Taiwan. Organic, non-GMO soybeans from Canada or the USA were soaked overnight in reverse osmosis (RO) water. They were then ground, and the juice (soy milk) was extracted. This grinding and extraction process was repeated three times to obtain moist crude okara with a moisture content of approximately 80-85%. The crude okara was baked in an oven dryer (Maido Co., Ltd, Taiwan) until it turned dark brown, then crushed to achieve a consistent particle size after cooling.

The dark brown dried okara was soaked in hot RO water (40 g/L) and boiled for 15 minutes. After cooling to room temperature, the solution was filtered using Whatman Grade 4 filter paper to remove the crude okara. The filtered SOE was then lyophilized into a powder using a freeze dryer. This SOE powder was reconstituted with double-distilled water and sterilized using a 0.2 µm syringe filter before being administered orally to the rats.

### ACLT-induced OA model

Eight-week-old Sprague Dawley (SD) rats (300-350 g) were purchased from the National Laboratory Animal Center (Taipei, Taiwan) and maintained in the animal center of China Medical University (Taichung, Taiwan). All animal procedures were approved by the Institutional Animal Care and Use Committee (IACUC). The ACLT surgery for OA induction was performed as previously described 23, 24. Rats in the control group (Control) underwent arthrotomy without transection of the ACL.

Two days after ACLT surgery, the rats were randomly divided into three groups and orally administered daily either 1 mL of RO water alone (the ACLT group), celecoxib at a dose of 50 mg/kg (the ACLT+Celecoxib group), or SOE at a dose of 40 mg/kg (the ACLT+SOE group), five days per week. After 6 weeks, following the final weight-bearing test, the rats were euthanized.

### Pain Behavior Weight Bearing Incapacitance Test

The static weight-bearing incapacitance test (Bioseb, Vitrolles, France) was conducted weekly to analyze spontaneous pain following ACLT, as previously described 25, 26. The difference in dynamic weight bearing (expressed in grams) over a 10-second period between the left and right hind limbs was measured using separate sensor plates. For each animal, three consecutive measurements were taken on each test day to calculate the average score. To account for variations in body weight among animals, the results are presented as a percentage of body weight.

### Micro-CT analysis

Micro-CT analysis was conducted 6 weeks post-ACLT surgery. The preparation of the right hind limb followed previously described protocols 27. Briefly, after excising the skin and muscle tissue, the femurs and tibias were fixed in 4% formaldehyde followed by 70% ethanol. The knee joints were scanned using the Bruker SkyScan 2211 micro-CT system (Kontich, Belgium). The scans were performed over a 180° rotation with a voltage of 70 kVp, a current of 290 µA, and a 0.5 mm aluminum filter to prevent beam-hardening artifacts. Image reconstruction was performed using InstaRecon® software (Bruker MicroCT, Kontich, Belgium), and 59 reoriented slices (0.5 mm each) were selected for further analysis. Manual regions of interest (ROIs) were drawn with an irregular contour in the subchondral trabecular bone region of the medial tibial plateau.

As previously described, image analysis for thresholding, ROI delineation, and various bone morphometric parameters, including bone mineral density (BMD), bone mineral content (BMC), bone volume/total volume (BV/TV), bone surface/total volume (BS/TV), trabecular number (Tb.N), trabecular thickness (Tb.Th), and trabecular separation (Tb.Sp), was performed using the Bruker MicroCT software (CTAn, version 1.7.1, Bruker, Kontich, Belgium) 26, 27.

### Histological analysis

Histopathological changes in OA tissue were analyzed using hematoxylin and eosin (H&E) and Safranin-O/Fast Green staining under an optical microscope, as previously reported 28, 29. Briefly, knee joint tissues fixed in 4% formaldehyde were decalcified with 10% EDTA in PBS for 14 days, followed by dehydration with increasing concentrations of ethanol (70% to 100%). The specimens were then embedded in paraffin blocks and cut into 5 µm-thick slices for histological staining. Structural changes in the cartilage of the central weight-bearing area of the medial tibial plateau were assessed using the Osteoarthritis Research Society International (OARSI) histopathology assessment system 30, which includes grading and staging scores to reflect the lesion depth and the extent of OA, respectively.

### Immunohistochemistry (IHC) Staining and Quantification Scoring

Immunohistochemistry analysis was conducted using the Leica Novolink Polymer Detection System (Leica Biosystems Inc., Buffalo Grove, IL, USA), as previously described 31. Briefly, tissue sections were pretreated with 3% hydrogen peroxide and incubated with 3% bovine serum albumin in PBS. The sections were then incubated with primary antibodies at 4°C overnight, followed by incubation with a peroxidase-conjugated secondary antibody at room temperature for 1 hour, and then stained with 3,3'-diaminobenzidine substrate.

Quantitative scoring, ranging from 0 to 7, represented both the intensity of the immunoreactive signals and the percentage of immunoreactive positive cells. The intensity of the immunoreactive signals was scored from 1 to 5 (from weak to strong). The percentage of immunoreactive cells was scored as follows: 0 = no signal; 1 = <10% positive cells; 2 = 10-29% positive cells; 3 = 30-59% positive cells; 4 = 60-100% positive cells. Scoring analysis was conducted by two independent assessors who were blinded to the treatment groups to minimize observer bias 30.

### Statistical Analysis

Statistical analyses for quantified results were conducted using GraphPad Prism version 5.0 software. Data are presented as the mean ± standard deviation (SD). The paired samples t-test and one-way ANOVA followed by Bonferroni post hoc testing were used to compare results between two groups and among more than two groups, respectively. Statistical significance was determined by a *p*-value < 0.05 in all cases.

## Results

### Oral SOE Does Not Affect Body Weight Growth Curve

**Figure 1:**
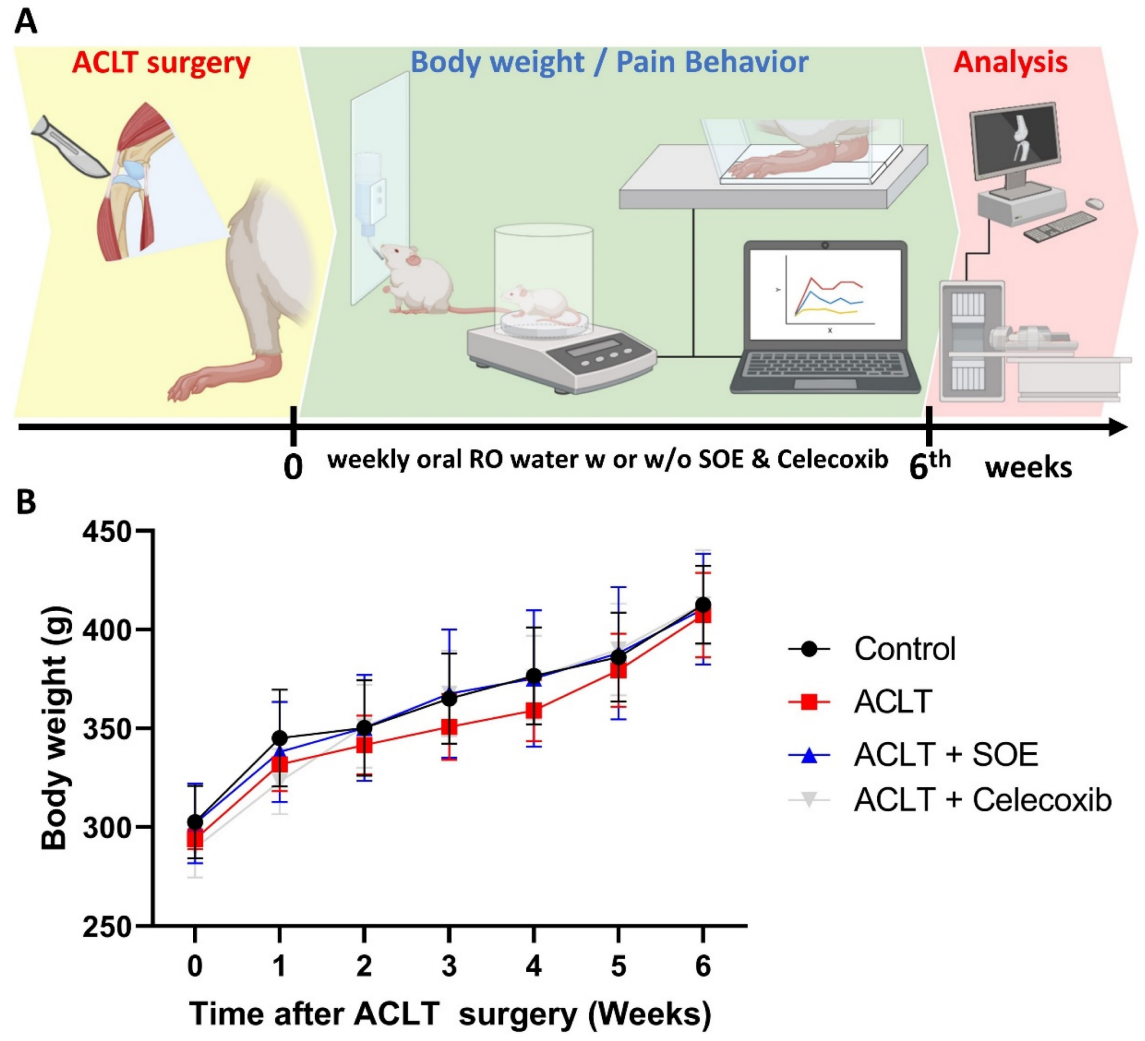
** (A) Schematic diagram of experiment design. (B) Growth curve of body weight throughout the experimental period.** No significant variations were observed between the study groups.

To investigate the protective effects of oral SOE, we utilized an ACLT-induced knee arthritis rat model and conducted pain behavior tests and histological analyses to explore the underlying mechanisms (Figure 1A). After a 3-5 -day habituation period in the animal center, the rats were randomly divided into four groups: arthrotomy without ACLT (Control; n=8), ACLT only (ACLT; n=8), ACLT with oral SOE (ACLT+SOE; n=8), and ACLT with celecoxib (ACLT+Celecoxib; n=8), the latter being an anti-COX-2 nonsteroidal anti-inflammatory drug that is a first-line treatment option for OA 32.

Body weights were recorded starting the day before surgery and were measured weekly until the rats were euthanized. During the experimental period, all groups showed a gradual increase in body weight, with no significant differences found among the groups (Figure 1B). This suggests that oral SOE does not have toxic side effects affecting body weight.

### Oral SOE Ameliorates OA Pain

**Figure 2:**
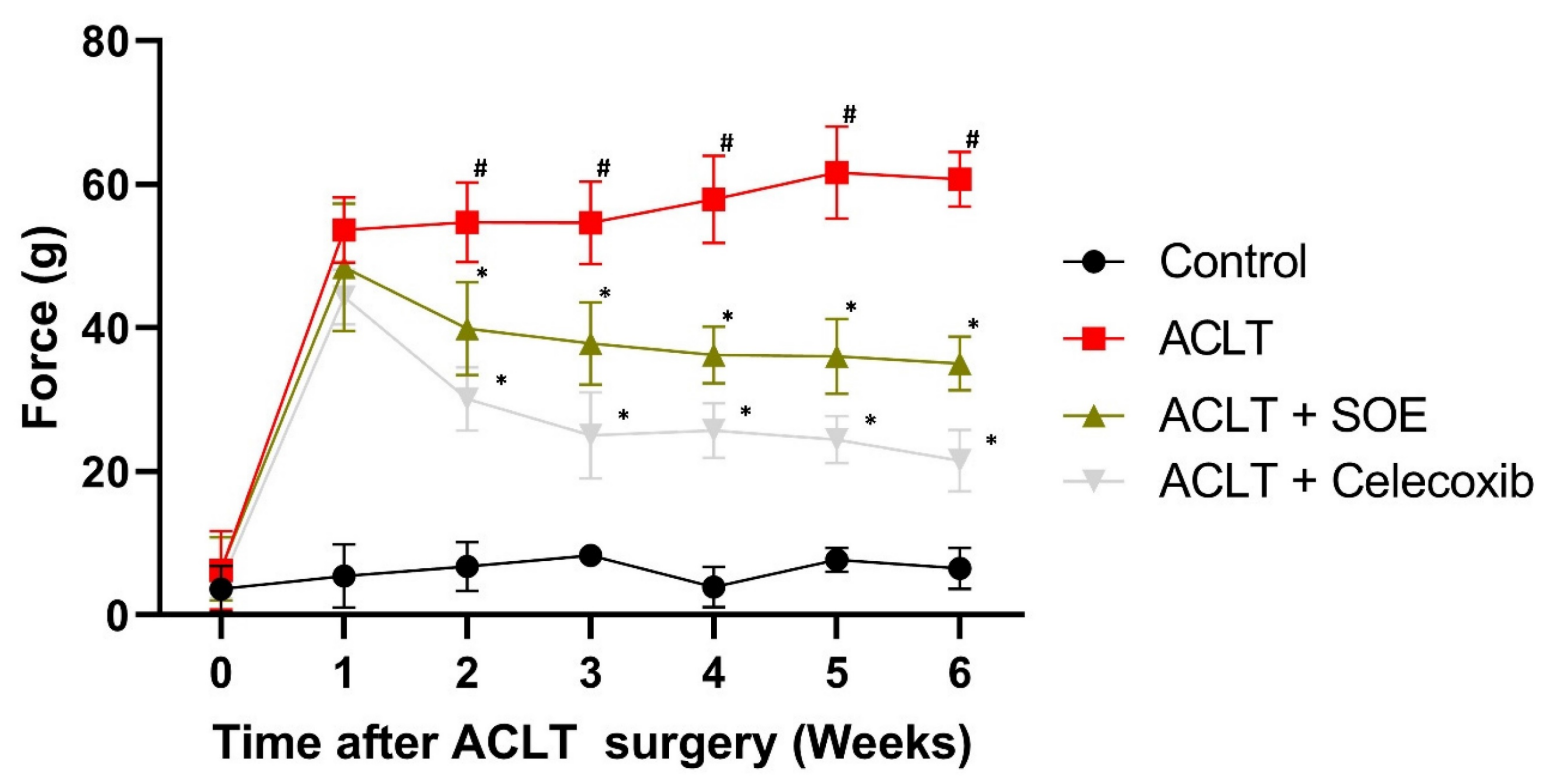
** Oral SOE decelerates ACLT-induced bone pain.** The pain behavior weight-bearing test suggests that rats of the ACLT+SOE and the positive control ACLT+Celecoxib groups were in less pain than ACLT group (*p < 0.05). #*p* < 0.05 ACLT vs. no surgery control group.

Pain behavior in the rats was evaluated using the static weight-bearing incapacitance test. In the first week post-surgery, all groups exhibited severe asymmetrical weight-bearing posture (Figure 2). This severe asymmetry worsened throughout the experimental period in the ACLT group. In contrast, the ACLT+SOE and ACLT+Celecoxib groups showed dramatic improvements in pain-associated behavior beginning in the second week post-surgery (Figure 2). These results suggest that oral SOE may effectively alleviate OA-related pain.

### Oral SOE Protects Osseous Damage in ACLT-induced OA

**Figure 3:**
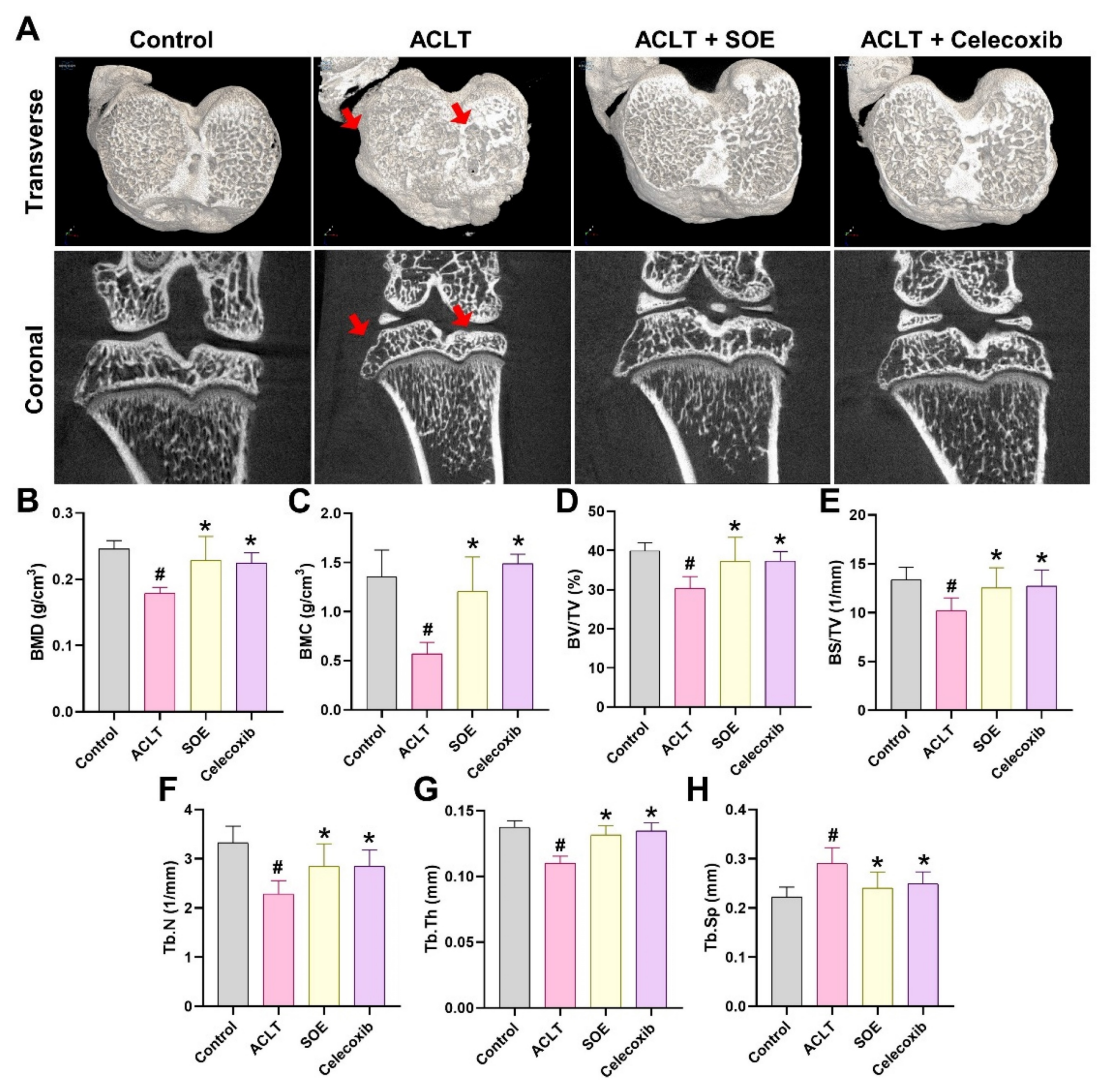
** Oral SOE ameliorates osseous damage in the ACLT-induced OA knee joint.** (A) Micro-CT images of rat right knee joints of control, ACLT and ACLT+SOE and ACLT+Celecoxib groups at transverse and coronal views. Red arrows indicate the bone loss. (B-H) Quantitative analyses of the BMD (B), BMC (C), (BV/TV (D), BS/TV (E), Tb.N (F), Tb.Th (G), and Tb.Sp (H) data in all study groups. # *p* < 0.05 vs. control group; * *p* < 0.05 vs. ACLT group.

Micro-CT analysis was conducted to assess changes in trabecular microarchitecture six weeks post-ACLT surgery. Significant bone damage was observed in the ACLT group compared to the Control group, confirming the OA lesion induced by ACLT surgery (Figure 3). Quantitative analysis showed loss of BMD, BMC, BV/TV, BS/TV, Tb.N, and Tb.Th, accompanied by an increase in Tb.Sp in the ACLT group (Figure 3B-H).

In contrast, notable improvements in bone architecture were observed in rats of the ACLT+SOE and ACLT+Celecoxib groups compared to the ACLT group (Figure 3). Quantitative data showed that there was no significant bone destruction in the ACLT+SOE and ACLT+Celecoxib groups when compared to the Control group (Figure 3B-H). These results suggest that oral SOE may protect against or ameliorate osseous damage in the OA knee.

### Oral SOE Protects against ACLT-Promoted Articular Cartilage Damage

**Figure 4:**
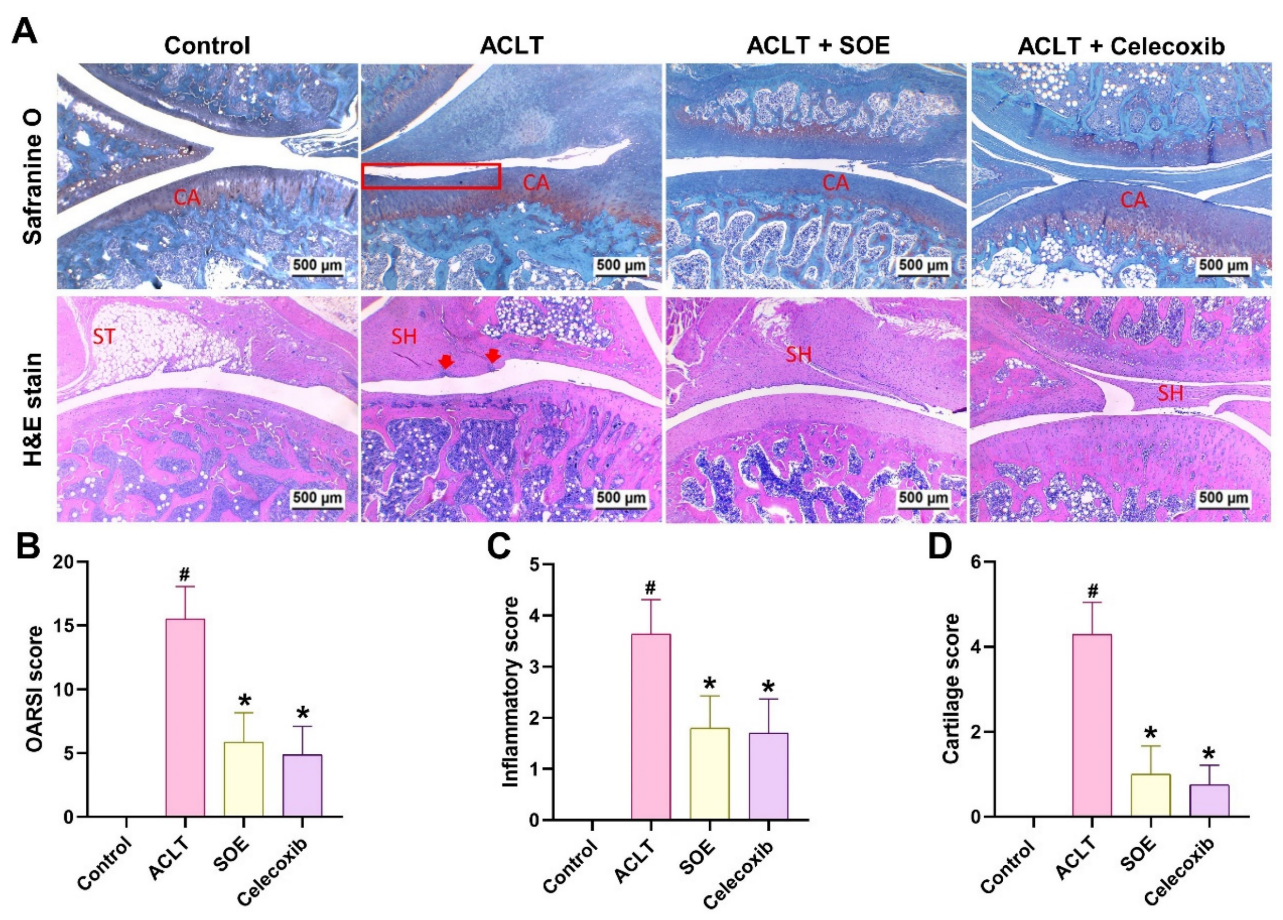
** Oral SOE ameliorates cartilage degradation in the ACLT-induced OA knee joint.** (A&B) Histopathological analysis of ACLT-induced OA knee articular cartilage damage in by Safranin-O/Fast Green (A) and H&E (B) staining. Scale bar = 500 μm. Assessment of osteoarthritic cartilage histopathology by OARSI (C), synovial membrane inflammation (D) and cartilage degeneration (E, arrow heads or red box) scores show less cartilage damage in oral SOE and celecoxib groups compared to ACLT group (* *p* < 0.05). # *p* < 0.05 vs. control group. Abbreviations: ST, synovial tissue; CA, cartilage; SH, synovial hyperplasia (arrows).

Histological analysis using H&E and Safranin-O/Fast Green staining revealed articular cartilage degradation and synovial lining hyperplasia in the ACLT group (Figure 4A). Quantification of OARSI scores, inflammation, and cartilage scores indicated that the ACLT+SOE and ACLT+Celecoxib groups exhibited fewer pathological changes in cartilage tissue and less synovial tissue hyperplasia compared to the ACLT group (Figure 4).

### Oral SOE Downregulates TNF-α, IL-1β, MMP-3 and Upregulates Aggrecan and COL2 Expression in OA Cartilage and Synovium

**Figure 5:**
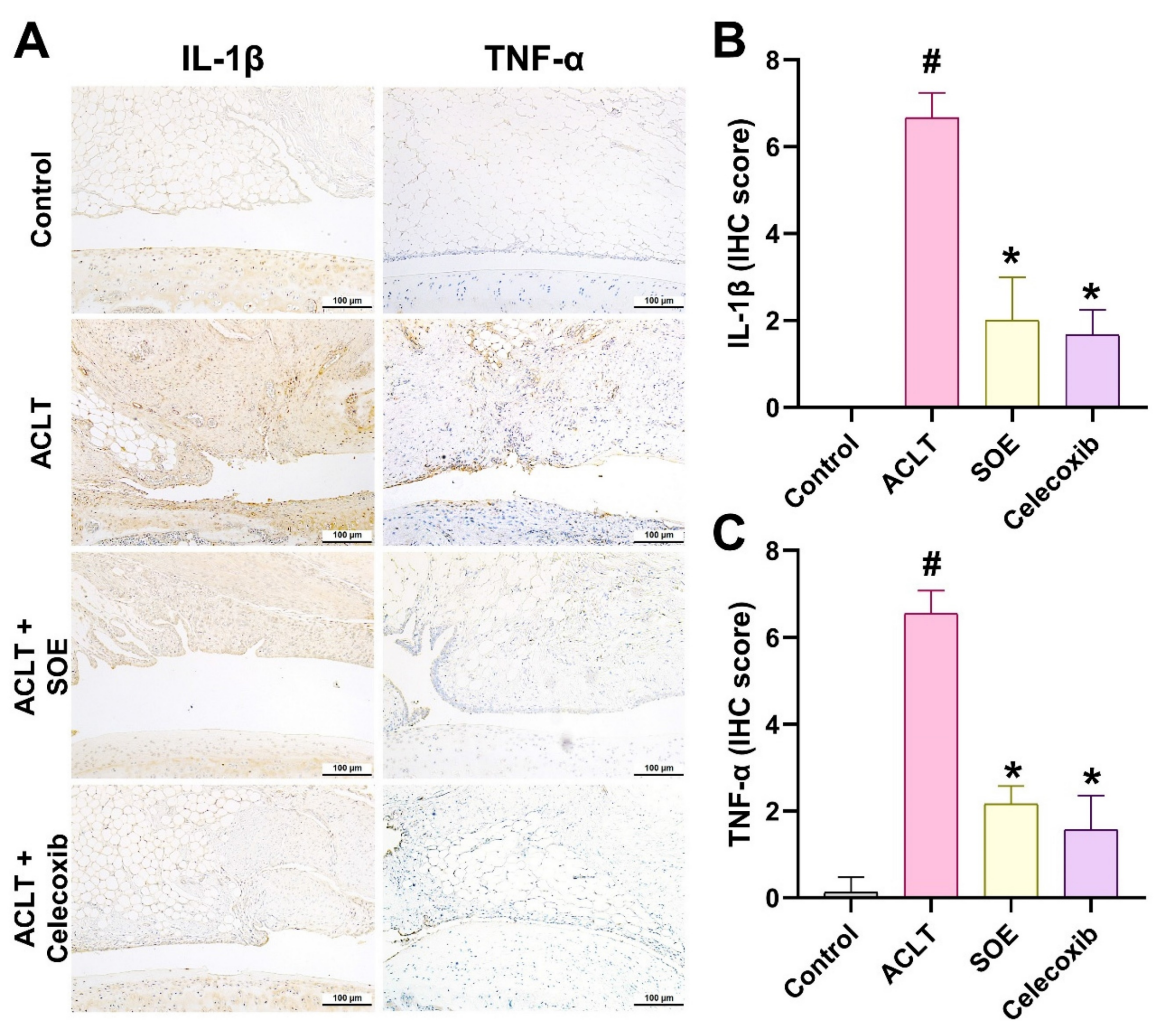
** Oral SOE suppress the induction of IL-1β and TNF-α in ACLT-induced OA articular cartilage.** Immuno-histochemistry analysis and scoring of IL-1β (A, B) and TNF-α (A, C) in rat right knee joint cartilage. Scale bar = 100 μm. # *p*<0.05 vs. control group. * *p*<0.05 vs. ACLT group.

IL-1β and TNF-α are key inflammatory mediators known to drive the progression of OA 8. IHC analysis revealed a significant elevation in the production of IL-1β and TNF-α in the synovial tissue of the ACLT group, indicating heightened inflammatory activity. This elevation was notably reduced in the ACLT+SOE and ACLT+Celecoxib groups (Figure 5).

**Figure 6:**
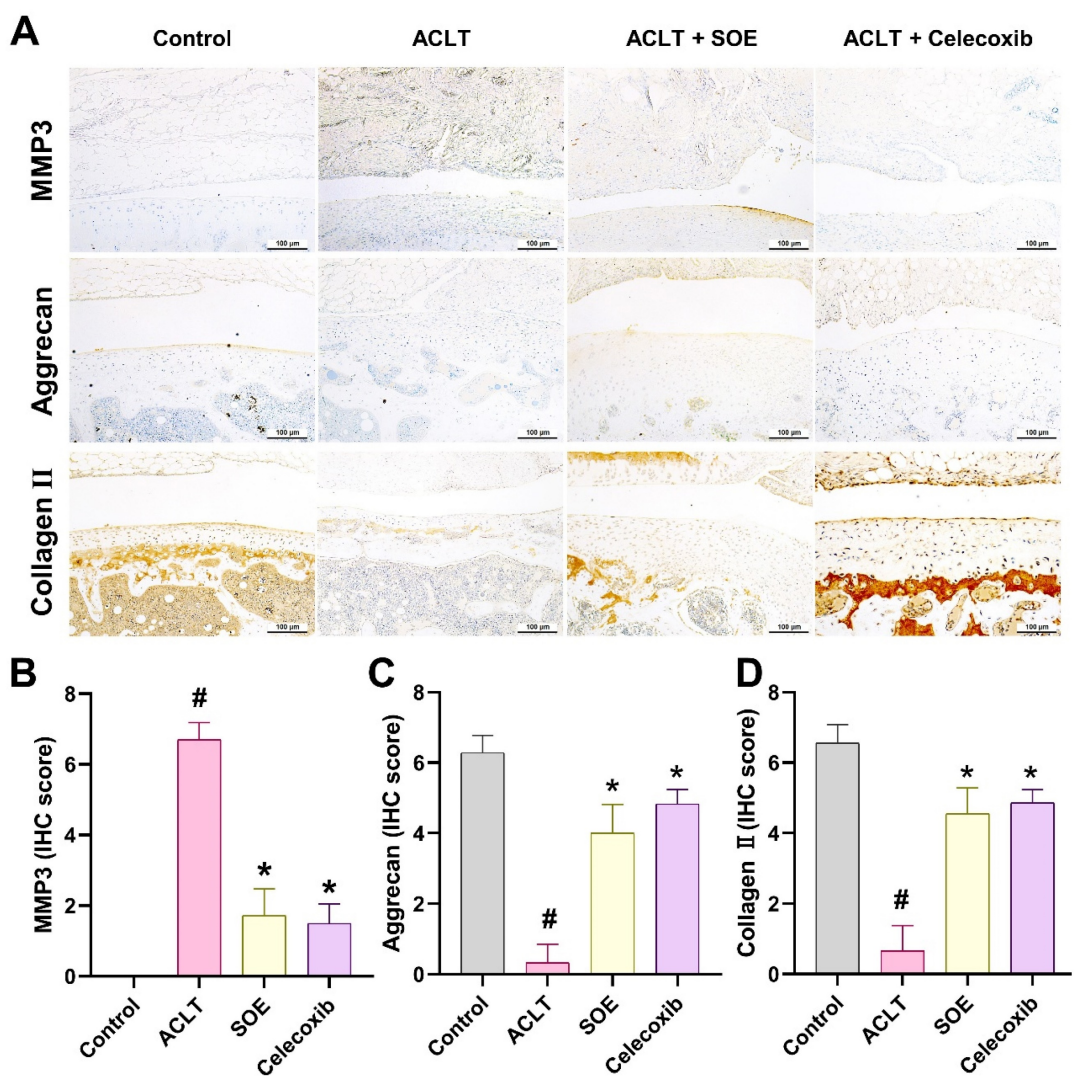
** Oral SOE reserve the expression of aggrecan and collagen II accompanying with suppression of MMP3 in ACLT-induced OA articular cartilage.** (A) Immuno-histochemistry analysis MMP3, aggrecan and collagen II in rat right knee joint cartilage. Scale bar = 100 μm. (B-D) Scoring of the immunosignals of MMP3 (B), aggrecan (C) and collagen II (D) in and scoring of MMP3, aggrecan and collagen II. # *p*<0.05 vs. control group. * *p*<0.05 vs. ACLT group.

Further assessment of cartilage metabolism was conducted through IHC staining for MMP-3, aggrecan, and collagen type II (COL2), which are critical components of articular cartilage. The ACLT+SOE and ACLT+Celecoxib groups exhibited lower levels of MMP-3, accompanied by higher levels of aggrecan and COL2, compared to the ACLT group (Figure 6). These findings suggest a protective effect of SOE and celecoxib on cartilage integrity in the ACLT-induced OA model.

## Discussion

This study demonstrates the potential of soybean okara as a beneficial food supplement for protecting against OA pain and progression. The protective effects were evidenced by the reduction in key inflammatory mediators, such as IL-1β, TNF-α, and MMP-3, resulting in less damage to articular cartilage and bone in a preclinical ACLT-induced OA rat model following oral administration of SOE. Therefore, we suggest that oral supplementation with SOE may be beneficial in the prevention and management of OA. In this study, celecoxib, an anti-COX-2 nonsteroidal anti-inflammatory drug and a first-line treatment option for OA 32, was used as a positive control. COX-2 inhibition is well-established for pain relief. However, it not only provides pain relief but also prevents cartilage degradation in preclinical OA models by inhibiting the production of inflammatory cytokines, which contribute to cartilage degradation and bone erosion.

Soybeans are a rich source of essential nutrients and bioactive compounds, including proteins, lipids, dietary fiber, and isoflavones, such as genistein and daidzein, which have been extensively documented for their protective effects against various chronic diseases, including OA 11, 12, 33-35. A clinical study comparing the effects of daily supplementation with 40 g of soy protein versus milk-based protein over three months showed that soy protein alleviated OA symptoms in men 33. Okara, the residue left after soybean processing, retains many of these beneficial nutrients and bioactive compounds, albeit in different proportions. Despite being a byproduct, okara remains rich in isoflavones and other compounds with relevant bioactivity 16, 21, 36. These retained components, particularly the isoflavones, likely contribute to the ability of SOE to mitigate inflammation and cartilage degradation, as demonstrated in our study 15, 16, 37. The preservation of these bioactive compounds and their effects in okara underscores its potential as a cost-effective, functional food supplement, offering protective benefits against chronic diseases similar to those of whole soybeans.

Genistein, a major soybean-derived isoflavone, is likely a key contributor to soybeans' protective effects against bone and cartilage diseases, including OA 15, 37. In human OA chondrocytes, genistein has been shown to reduce IL-1β-promoted production of NOS2, COX-2, and MMPs through the activation of the NRF2/HO-1 pathway. This pathway is associated with inhibition of oxidative stress, and its activation by genistein suggests a chondroprotective effect 37. Furthermore, both *in vitro* and *in vivo* studies have shown genistein's anti-apoptotic effects on inflammatory OA chondrocytes and on rat OA articular cartilage 37, 38. Genistein's ability to reduce IL-1β, TNF-α, and caspase-3 levels, while increasing collagen type II and aggrecan expression, aligns with the therapeutic effects observed in our study, suggesting that genistein may play a crucial role in the protective effects of oral SOE 38.

The protective effects of SOE may also be linked to the estrogenic properties of soybean isoflavones 39. Estrogen receptors are present in articular cartilage, bone, synovial tissue, and joint ligaments. Estrogen deficiency, particularly post-menopause, has been associated with an increased incidence of OA in women. In ovariectomized rats, soybean isoflavones have been shown to protect against OA 35. However, the protective effects observed in this study were in male rats, suggesting that the estrogenic effects of SOE, if present, were minimal or not significant in this model. This observation highlights the potential of SOE as a therapeutic option for OA that may act independently of estrogenic mechanisms.

Recent research has increasingly focused on soy-derived bioactive peptides, which are generated through the enzymatic hydrolysis of soybean proteins 12, 40, 41. These peptides exhibit a wide range of physiological functions, including hypolipidemic, immunomodulatory, anticancer, antioxidant, and anti-inflammatory effects, highlighting their significant health benefits 12, 40, 41. Among these, lunasin, a promising soybean-derived bioactive peptide, has been found to suppress IL-1β-promoted expression of MMP-3 and MMP-13, prevent the decrease in TIMP-1 and TIMP-2, and inhibit the reduction in COL2 in chondrocytes 42. Lunasin is a heat-stable peptide composed of 43 amino acids and is recognized for its antioxidative, anti-inflammatory, and anticancer properties 43. Given these characteristics, it is plausible that lunasin is one of the bioactive components contributing to the chondroprotective effects observed with SOE in our study. Although bioactive components, such as isoflavones (e.g., genistein) and peptides (e.g., lunasin), contribute to the anti-OA effects of SOE, we did not perform quantitative analysis to identify the major bioactive compounds in SOE. Further investigation is needed to quantify the bioactive components in SOE.

In conclusion, our data provide the first evidence that okara, rich in soybean-derived bioactive compounds, offers protective effects against OA. These findings suggest that okara, traditionally considered a byproduct of soybean processing, retains beneficial properties similar to those of soybeans and may be effective in the prevention of chronic diseases and the promotion of overall health. Additionally, the preservation of okara using an oven dryer effectively extends its shelf life without compromising its bioactive properties. The results of this report highlight the potential health applications of okara, enhancing its economic value and offering a sustainable approach to improving human health.
